# Supplementary material for: Label-Free Digital Holotomography Reveals Ibuprofen-Induced Morphological Changes to Red Blood Cells
Source: ACS Nanosci Au. 2023 Apr 5;3(3):241–55. doi: 10.1021/acsnanoscienceau.3c00004 (PMC10288613; doi:10.1021/acsnanoscienceau.3c00004)
Supplement: Supplementary file 1 — ng3c00004_si_001.pdf [file ng3c00004_si_001.pdf]

Supporting Information

Label-free digital holotomography reveals ibuprofen-induced morphological changes to red blood cells.

Talia Bergaglio[a,b], Shayon Bhattacharya[c], Damien Thompson[c]*, Peter Niraj Nirmalraj[a]*

[a]Transport at Nanoscale Interfaces Laboratory, Swiss Federal Laboratories for Materials Science and Technology, Dübendorf CH-8600, Switzerland; [b]Graduate School for Cellular and Biomedical Sciences, University of Bern, CH-3012, Switzerland; [c]Department of Physics, Bernal Institute, University of Limerick, Limerick V94T9PX, Ireland.

[1]To whom correspondence may be addressed: peter.nirmalraj@empa.ch and damien.thompson@ul.ie

This PDF file includes:

Section S1
Figures S1 to S15
Tables S1 to S6
Legends for Movies S1 to S7
SI References

Other supporting materials for this manuscript include the following:

Movies S1 to S7

Section S1. Molecular dynamics model details

S1.1 RBC membrane lipid bilayer model. We modelled the RBC membrane lipid bilayer based on the *in silico* lipid composition of the model erythrocyte membrane in ref. (1), which have been previously experimentally validated. The membrane model was built using CHARMM-GUI (2, 3) web-interface and is composed of 20% and 20% cholesterol (CHL), 11% and 25% Palmitoyl Oleoyl PhosphoCholine (POPC), 38% and 10% Palmitoyl Oleoyl PhosphoEthanolamine (POPE), 22% and 0% Palmitoyl Oleoyl PhosphoSerine (POPS), and 9% and 35% Stearoyl SphingoMyelin (SSM) in the inner and outer leaflets, respectively (see Table S4 and Fig. S12*A*), with 200 lipid molecules in each leaflet. The surface area of the lipid bilayer was 10.5 X 10.5 nm^2 (Fig. S12*B*), large enough to study adsorption of ibuprofen aggregates on membrane surface.

S1.2 Preparation of the ibuprofen-lipid systems and molecular dynamics simulations. Molecular Dynamics (MD) simulations were performed using Gromacs 2018.4 (4) software. The ibuprofen molecules and RBC lipid bilayer were represented by CHARMM General force field (5, 6) (CGenFF) and CHARMM36m (7) force field parameters, respectively. *Five* different ibuprofen aggregates were studied on top of RBC membrane bilayer – *(1)* single molecule of ibuprofen (Fig. S12*C*), preformed aggregates of *(2)* 80 ibuprofen molecules, representing very low concentration, and *(3)* 100 ibuprofen molecules representing low concentration, but higher than 80-molecule aggregate, densely packed box of ibuprofen containing 1903 molecules under *(4)* isothermal-isobaric ensemble (NPT) conditions at constant pressure, where the volume of the system is adjusted during simulation representing high concentration, and *(5)* canonical ensemble (NVT) at constant volume to model very high concentration of ibuprofen aggregates. System *(1)* will henceforth be regarded as “single ibu”, system *(2)* as “low ibu conc. I”, system *(3)* as “low ibu conc. II”, system *(4)* as “high ibu conc. I”, and system *(5)* as “high ibu conc. II”. The preformed aggregates of ibuprofen (80 and 100 molecules) were modelled by running a MD simulation of randomly dispersed molecules of ibuprofen in water with counterions (Na^+ and Cl^-) (see Fig. S12*D*). The ibuprofen aggregates formed instantly within 1 ns dynamics (Fig. S12*D*) due to strong hydrophobic intermolecular forces. The starting configuration of all five systems are shown in Figs. S12*A–E*. All ibuprofen–membrane complexes were solvated by filling the area above and below the membrane with water molecules represented by the modified TIP3P water model (8), creating a >20-Å thick water layer above the ibuprofen and below the membrane to mimic bulk solvation in the z-plane. Each simulation cell was neutralized by adding the appropriate number of counterions. After 5000 steps of energy minimization, each system was equilibrated over six consecutive steps (100 ps each), with the values of the force constants of position and dihedral restraints of lipids gradually decreased from 1000 to 0 (the unit for position and dihedral restraints are kJ/(mol.nm^2) and kJ/(mol.rad^2), respectively). During equilibration, the Berendsen thermostat and barostat were applied to maintain the temperature at 310 K and pressure at 1 atm. Semi-isotropic pressure coupling was applied to allow the lipid bilayer to fluctuate in the xy plane independent of the z-axis. For the production run, the Velocity rescaling thermostat and Parrinello-Rahman barostat were applied. Long-range electrostatic interactions were treated using the particle-mesh Ewald (PME) method. The time step used in our MD simulations is 2 fs, and the structures were saved every 100 ps during 0.1 μs of production dynamics.

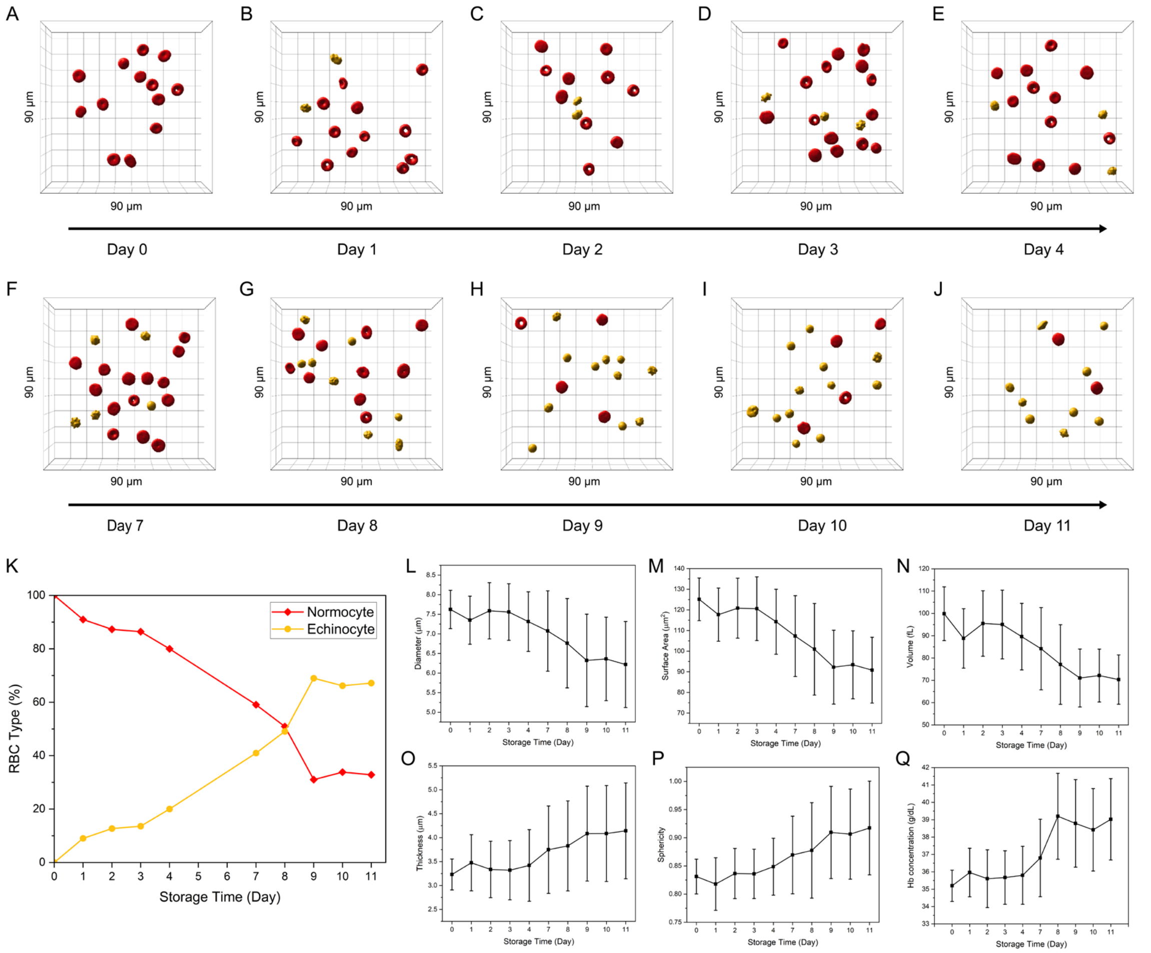

Fig. S1. Evolution of RBC quality over 11-days of storage time. 3D rendering of healthy RBCs diluted in PBS and stored at 4°C on (*A*) day 0, (*B*) day1, (*C*) day 2, (*D*) day 3, (*E*) day 4, (*F*) day 7, (*G*) day 8, (*H*) day 9, (*I*) day 10, (*J*) day 11. (*K*) Percentage of normocytes and echinocytes in the same blood solution over 11 days. (*L*)-(*Q*) Quantification of RBC morphological parameters over storage time: diameter, surface area, volume, thickness, sphericity and Hb concentration. Error bars depict the standard deviation. Field of view 90x90x30 µm.

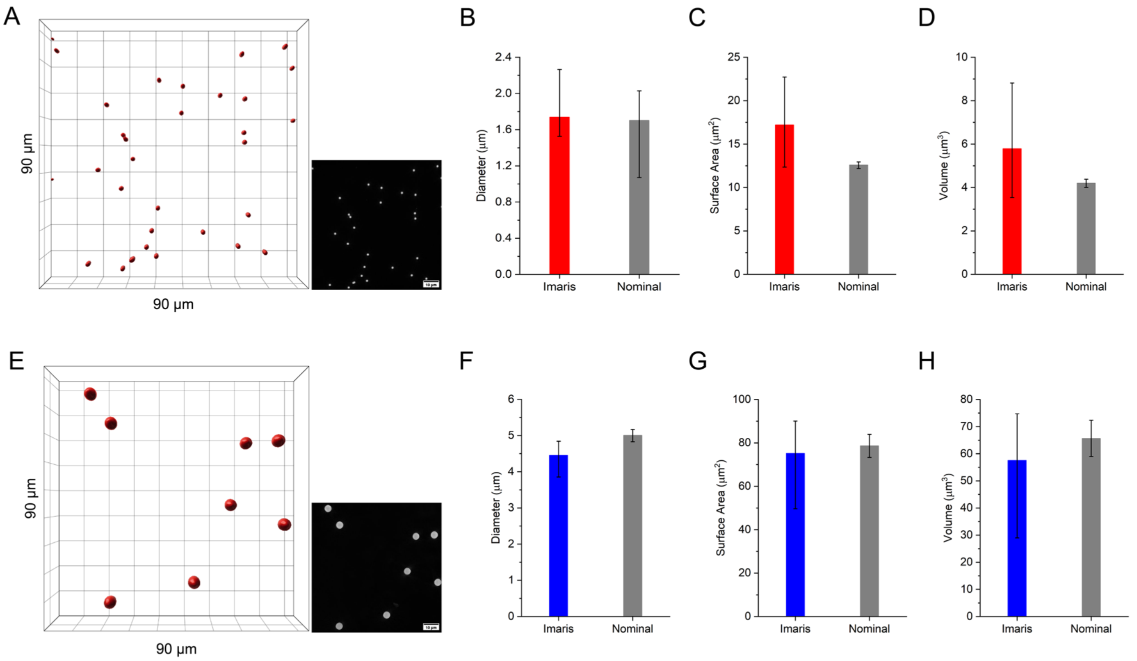

Fig. S2. Benchmark for the quantification of the morphological parameters using micro-particles based on silicon dioxide (Merk Millipore). (*A*) 3D rendering of 2 µm beads with corresponding RI tomogram in the inset, obtained with DHTM. Comparison of the quantification of (*B*) the diameter, (*C*) the surface area and (*D*) the volume, between the Imaris-based image analysis and the nominal values provided by the manufacturer. (*E*) 3D rendering of 5 µm beads with corresponding RI tomogram in the inset, obtained with DHTM. (*F*) Comparison of the quantification of the diameter, (*G*) the surface area and (*H*) the volume, between the Imaris-based image analysis and the nominal values provided by the manufacturer. Error bars depict the minimum and maximum values. Field of view 90x90x30 µm.

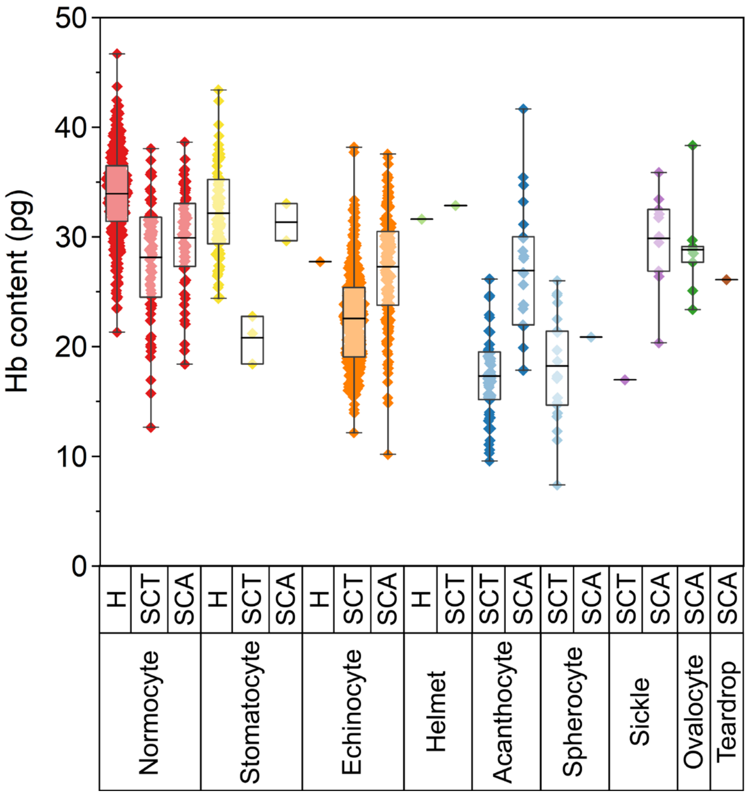

Fig. S3. Quantification of Hb content in healthy, SCT and SCA RBC populations based on 3D tomograms. Single cell level comparison between ML-based classified RBC types in healthy, SCT and SCA samples. Bars indicate mean values plus minimum and maximum values of all counted cells in each group.

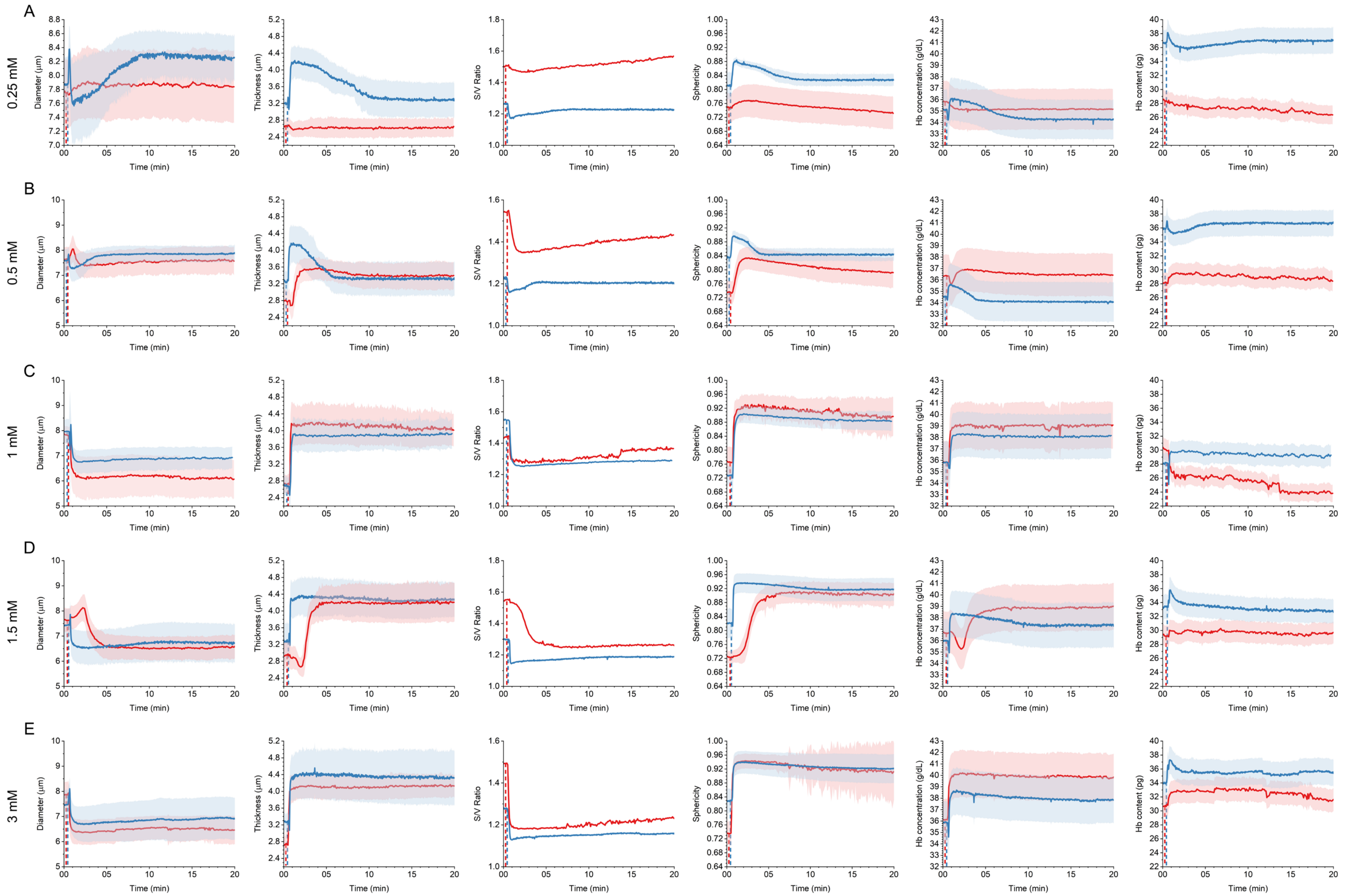
A
0.25 mM
B
0.5 mM
C
1 mM
D
1.5 mM
E
3 mM
Diameter (µm)
Thickness (µm)
S/V Ratio
Sphericity
Hb concentration (g/dL)
Hb content (pg)
Time (min)

Fig. S4. Quantification of RBC morphological changes in diameter, thickness surface area to volume ratio (S/V Ratio), sphericity, Hb concentration and Hb content upon exposure to different concentrations of ibuprofen (0.25 mM, 0.5 mM, 1 mM, 1.5 mM and 3mM) during a 20-minute time-lapse. (*A*) 0.25 mM, (*B*) 0.5 mM, (*C*) 1 mM, (*D*) 1.5 mM and (*E*) 3 mM ibuprofen concentration. Blue line = field of view 1; red line = field of view 2.

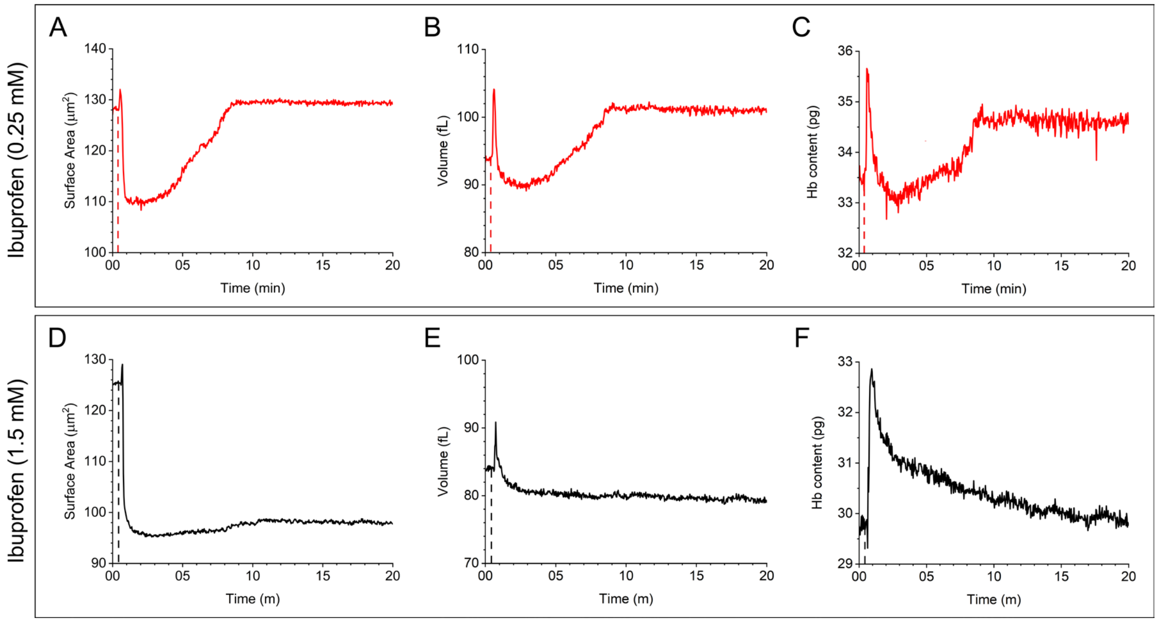

Fig. S5. Quantification of RBC morphological changes in surface area, volume and Hb content upon exposure to low and high concentrations of ibuprofen during a 20-minute time-lapse. (*A-C*) Time-dependent changes to surface area, volume and Hb content of a single RBC treated with 0.25 mM ibuprofen. (*D-F*) Time-dependent changes to surface area, volume and Hb content of a single RBC treated with 1.5 mM ibuprofen.

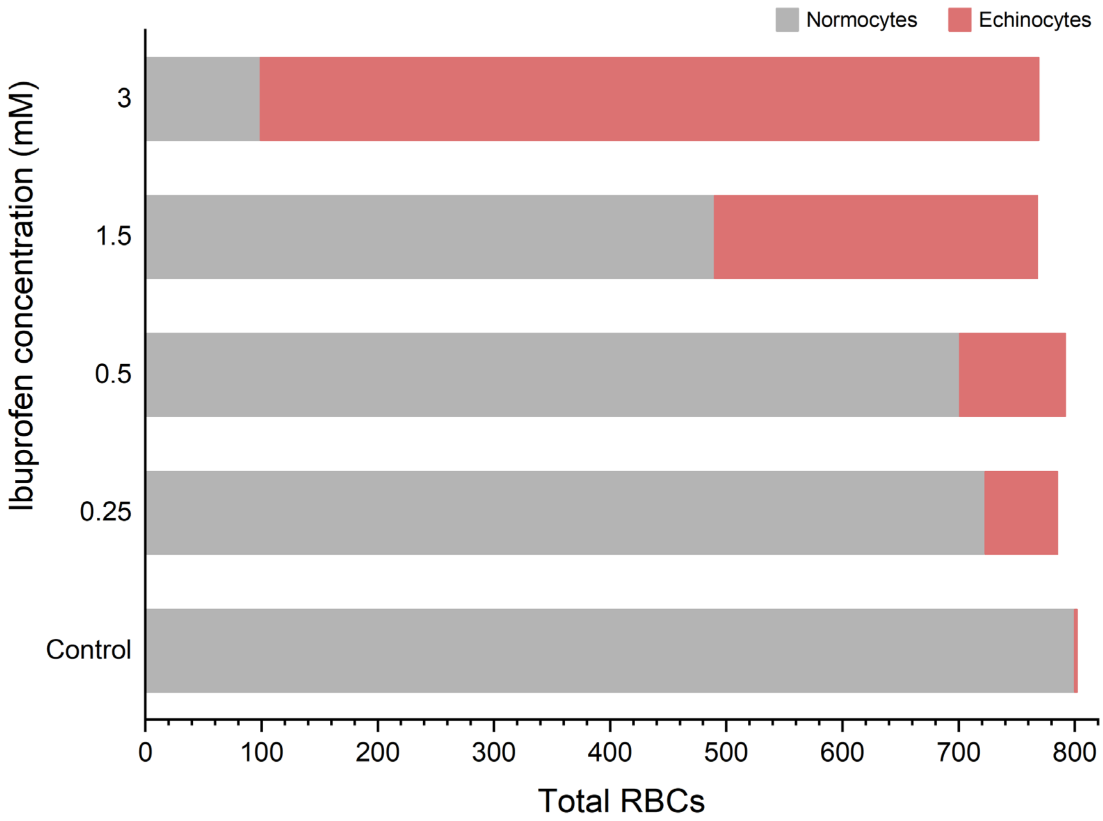

Fig. S6. Ratio of normocytes and echinocytes in blood incubated with 0.25 mM, 0.5 mM, 1.5 mM and 3 mM ibuprofen concentrations for 1.5 hours at 37°C.

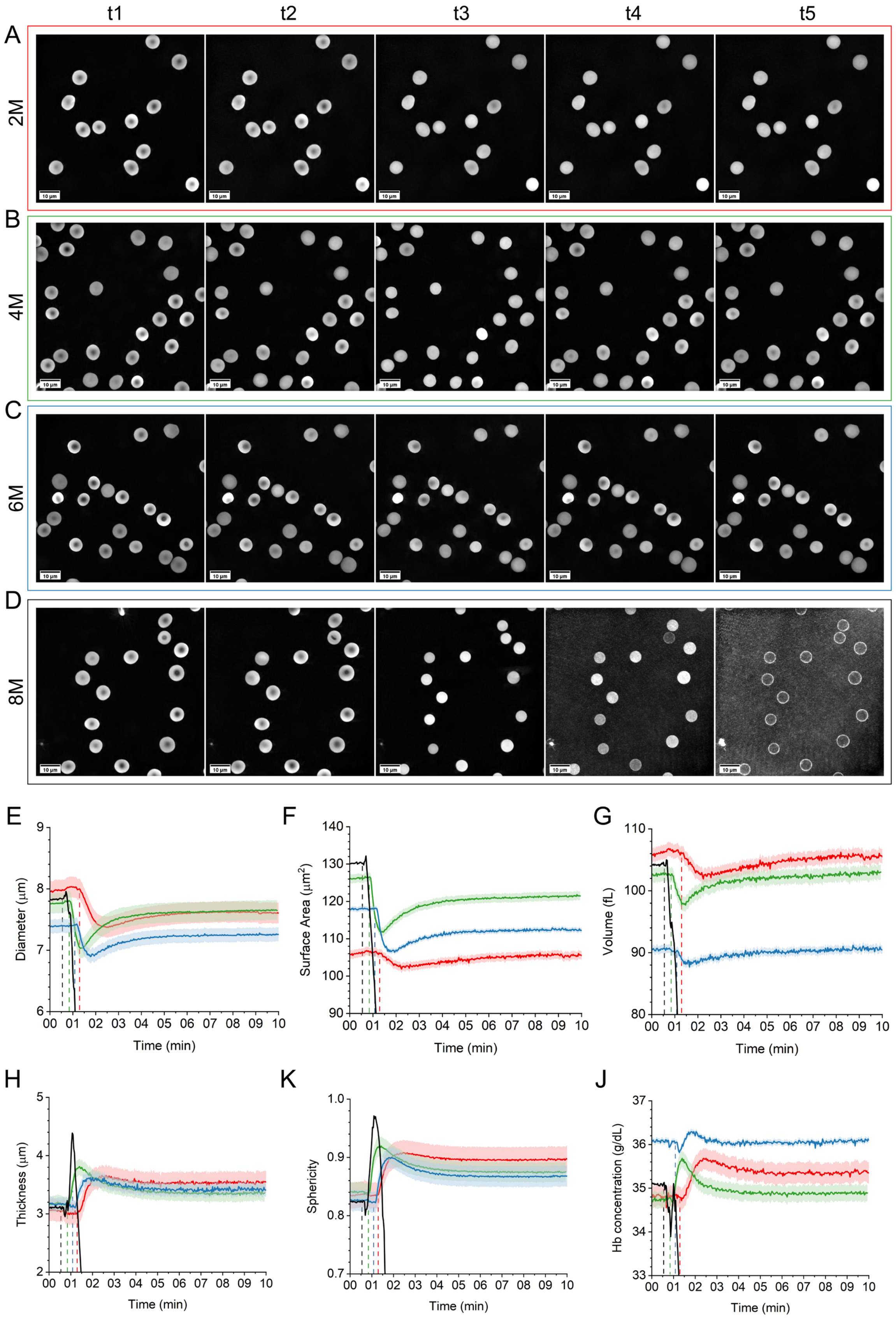
t1
t2
t3
t4
t5
A
2M
B
4M
C
6M
D
8M
E
Diameter (µm)
Time (min)
F
Surface Area (µm2)
Time (min)
G
Volume (fL)
Time (min)
H
Thickness (µm)
Time (min)
K
Sphericity
Time (min)
J
Hb concentration (g/dL)
Time (min)

Fig. S7. 3D holo-tomographic imaging of RBCs treated with urea at varying concentrations, during a 10-minute time-lapse. (*A*) 3D RI tomograms of RBCs treated with 2M urea, resulting in mild spherocytosis (*t1*: 0 s; *t2*: 1:24 min, urea added; *t3*: 1:48 min; *t4*: 5:20 min; *t5*: 10 min). (*B*) 3D RI tomograms of RBCs treated with 4M urea, resulting in mild spherocytosis (*t1*: 0 s; *t2*: 1:02 min, urea added; *t3*: 1:26 min; *t4*: 4:58 min; *t5*: 10 min). (*C*) 3D RI tomograms of RBCs treated with 6M urea, resulting in mild spherocytosis (*t1*: 0 s; *t2*: 1:22 min, urea added; *t3*: 1:46 min; *t4*: 5:18 min; *t5*: 10 min). (*D*) 3D RI tomograms of RBCs treated with 8M urea, resulting in spherocytosis and cell lysis, with formation of ghost cells (*t1*: 0 s; *t2*: 48 s, urea added; *t3*: 1:08 min; *t4*: 2:20 min; *t5*: 10 min). (*E*-*J*) Quantification of time-dependent morphological parameters in urea-treated RBCs, (*E*) diameter, (*F*) surface area, (*G*) volume, (*H*) thickness, (*K*) sphericity and (*J*) Hb concentration (red = 2M; green = 4M; blue = 6M; black = 8M). Field of view 90x90x30 µm.

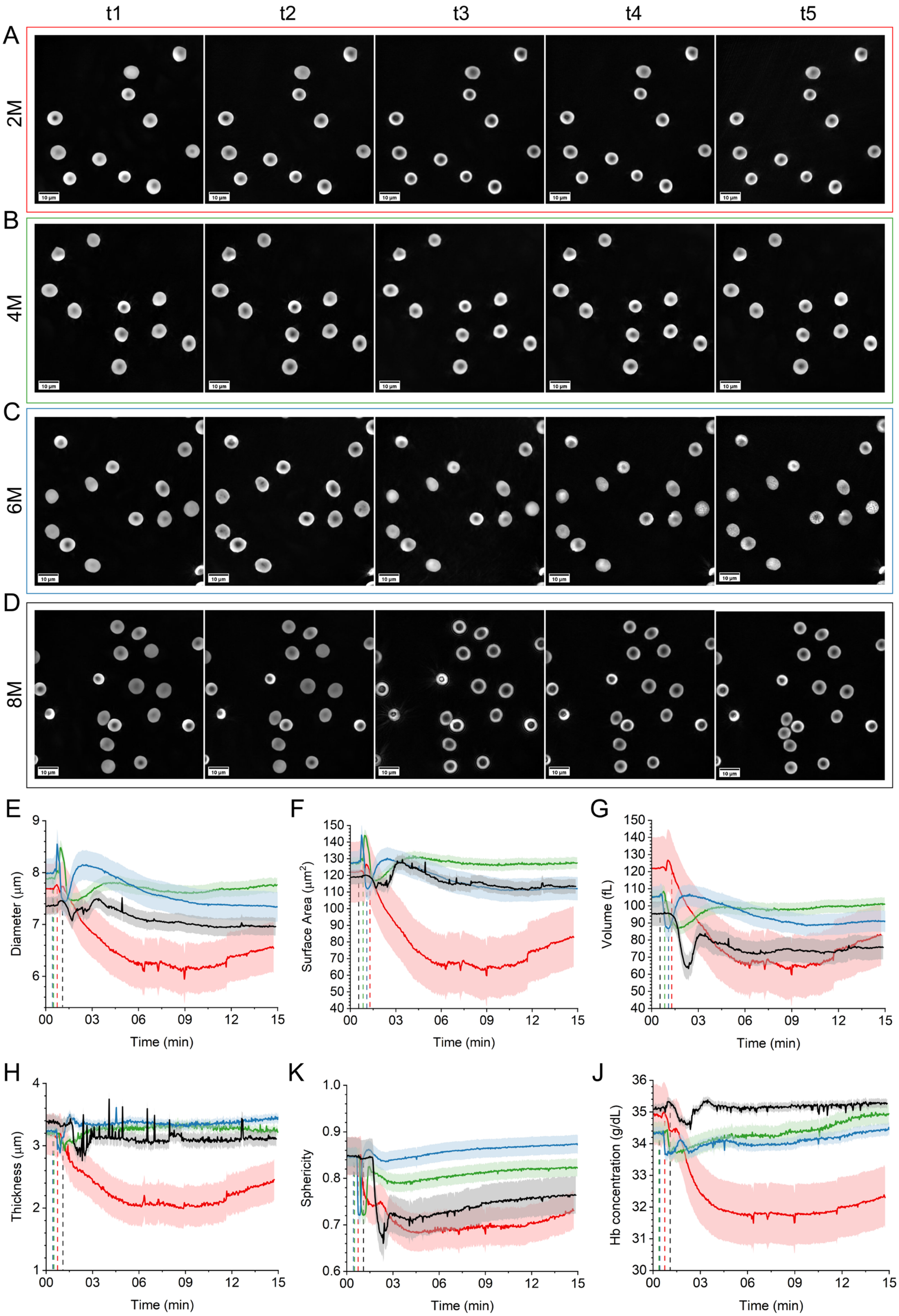
t1
t2
t3
t4
t5
A
B
C
D
2M
4M
6M
8M
E
F
G
H
K
J
Diameter (μm)
Surface Area (μm2)
Volume (fL)
Thickness (μm)
Sphericity
Hb concentration (g/dL)
Time (min)

Fig. S8. 3D holo-tomographic imaging of RBCs treated with H_2O_2 at varying concentrations, during a 10-minute time-lapse. (*A*) 3D RI tomograms of RBCs treated with 2M H_2O_2, resulting in mild stomatocytosis (*t1*: 0 s; *t2*: 1:02 min, H_2O_2 added; *t3*: 1:48 min; *t4*: 7:44 min; *t5*: 15 min). (*B*) 3D RI tomograms of RBCs treated with 4M H_2O_2, resulting in mild stomatocytosis (*t1*: 0 s; *t2*: 54 s, H_2O_2 added; *t3*: 1:34 min; *t4*: 6:34 min; *t5*: 15 min). (*C*) 3D RI tomograms of RBCs treated with 6M H_2O_2, resulting in mild stomatocytosis (*t1*: 0 s; *t2*: 44 s, H_2O_2 added; *t3*: 1:28 min; *t4*: 6:24 min; *t5*: 15 min). (*D*) 3D RI tomograms of RBCs treated with 8M H_2O_2, resulting in more pronounced stomatocytosis (*t1*: 0 s; *t2*: 1:40 min, H_2O_2 added; *t3*: 2:20 min; *t4*: 7:20 min; *t5*: 15 min). (*E*-*J*) Quantification of time-dependent morphological parameters in urea-treated RBCs, (*E*) diameter, (*F*) surface area, (*G*) volume, (*H*) thickness, (*K*) sphericity and (*J*) Hb concentration (red = 2M; green = 4M; blue = 6M; black = 8M). Field of view 90x90x30 μm.

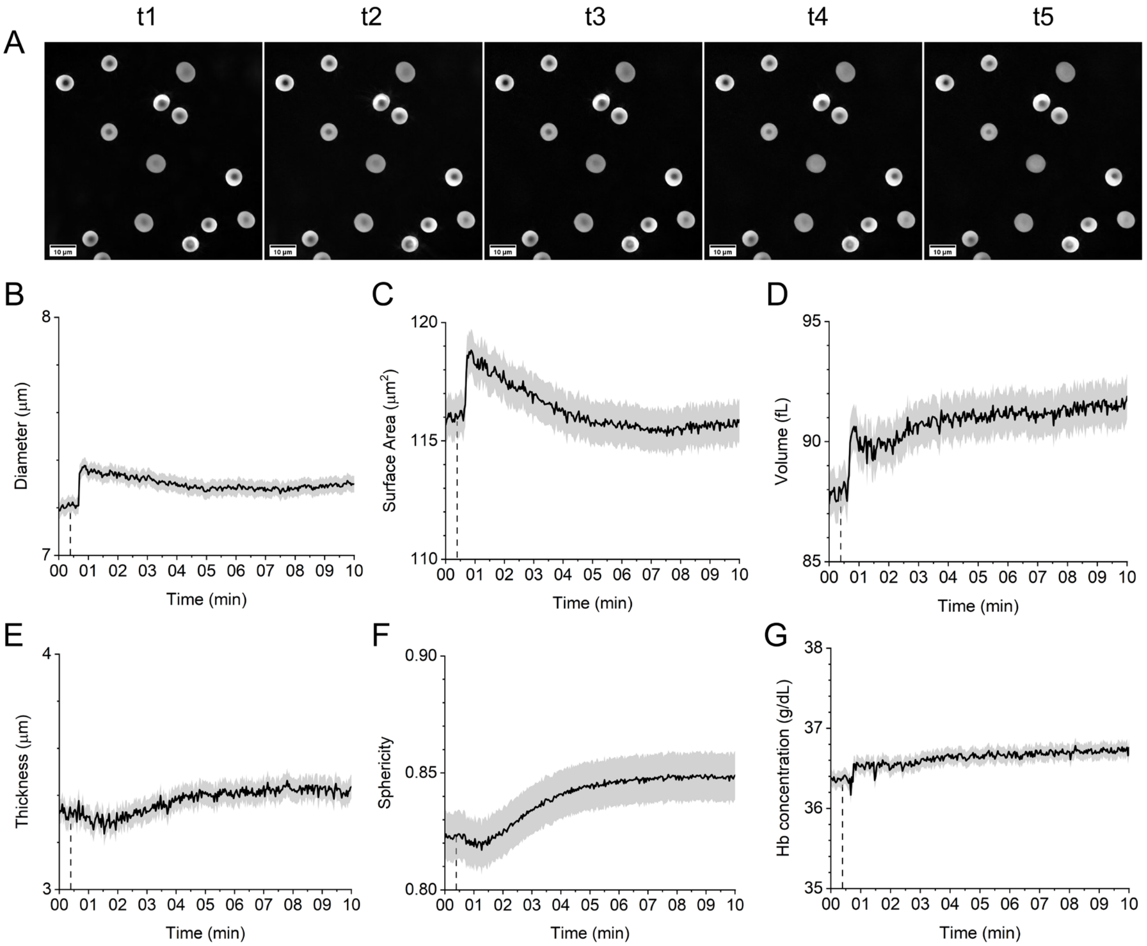

Fig. S9. 3D holo-tomographic imaging of RBCs treated with ddH_2O, during a 10-minute time-lapse. (*A*) 3D RI tomograms of RBCs treated with ddH_2O_2, resulting in no morphological alteration (*t1*: 0 s; *t2*: 44 s, ddH_2O added; *t3*: 1:04 min; *t4*: 4:00 min; *t5*: 10 min). (*B*-*G*) Quantification of time-dependent morphological parameters in urea-treated RBCs, (*B*) diameter, (*C*) surface area, (*D*) volume, (*E*) thickness, (*F*) sphericity and (*G*) Hb concentration. Field of view 90x90x30 μm.

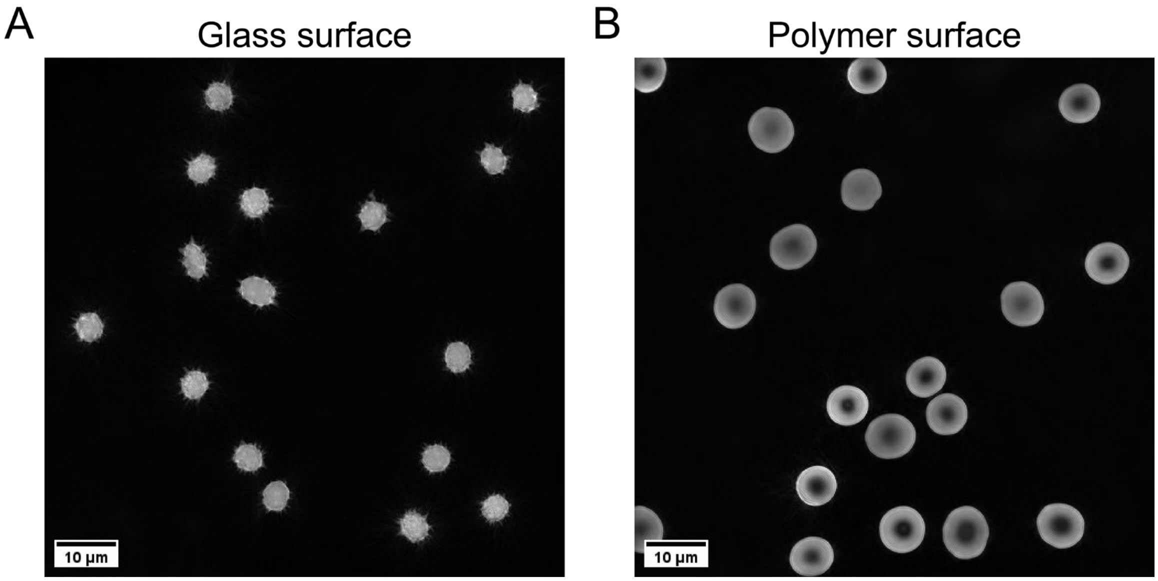

Fig. S10. Effect of petri dish surface used to image blood solutions with DHTM. (*A*) Echinocytosis resulting from RBCs contact with a glass surface. (*B*) Polymer-coated surface resulting in unaltered RBC morphology.

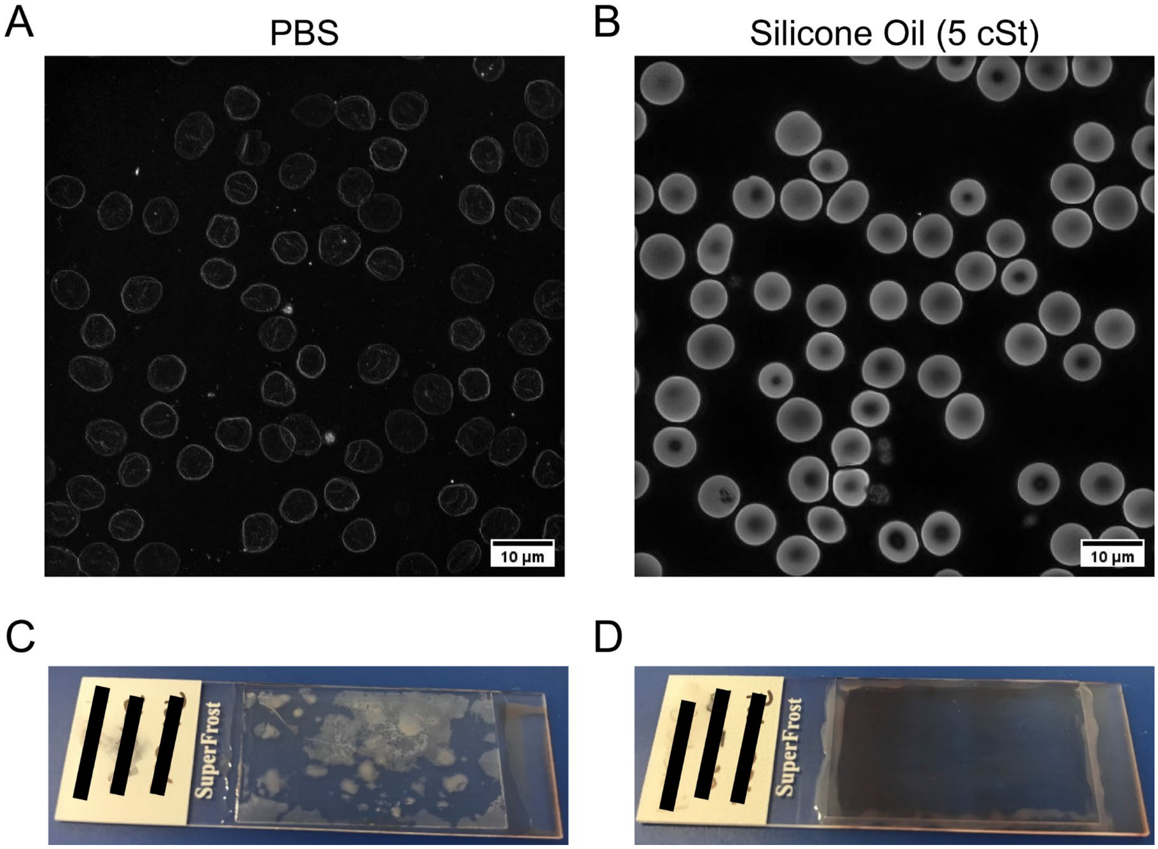

Fig. S11. Comparison between PBS and silicone oil (5 cSt) coating for optimal blood smear imaging with DHTM. (*A*) RI tomogram of healthy RBCs from a blood smear coated with PBS. (*B*) RI tomogram of healthy RBCs from a blood smear coated with silicone oil (5 cSt). (*C*) Example of storage artifacts in a blood smear coated with PBS and sealed with a coverslip and nail varnish after 4 days storage at room temperature. (*D*) Example of absence of storage artifacts in a blood smear coated with silicone oil (5 cSt) and sealed with a coverslip and nail varnish after 4 days storage at room temperature.

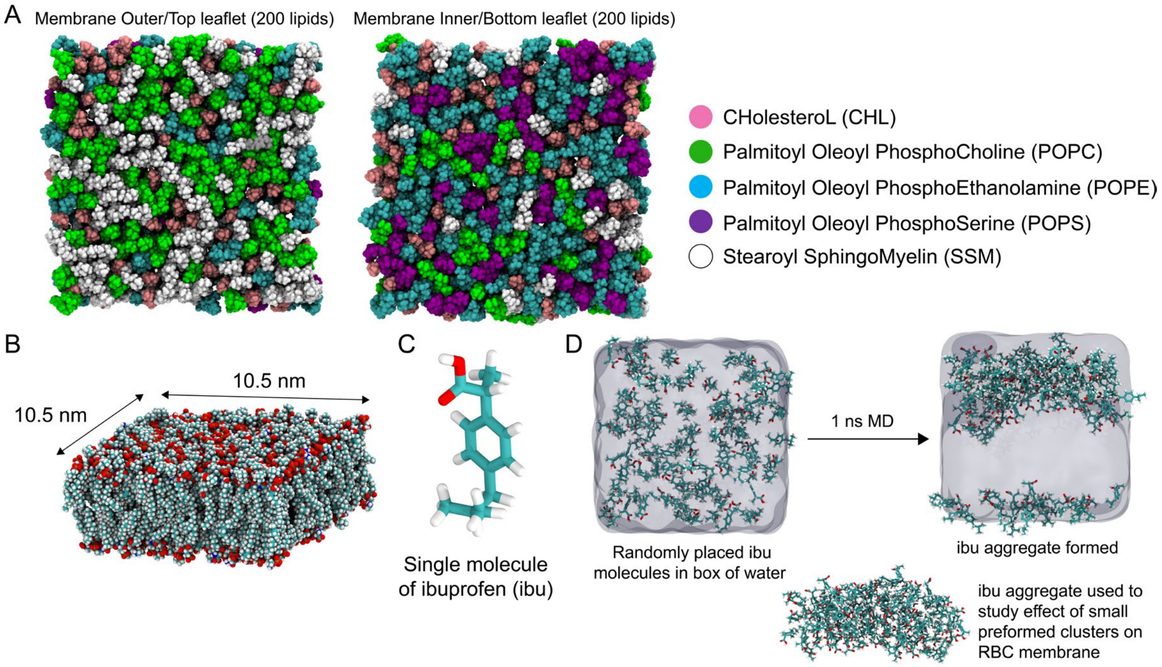

Fig. S12. *(A)* Model RBC lipid bilayer composition at the outer/top and inner/bottom leaflets used in this study also showing the *(B)* membrane dimensions in the xy-plane. *(C)* Stick representation of a single molecule of ibuprofen (ibu). *(D)* Preformed aggregates of ibuprofen molecules (at low concentration) obtained in this study from 1 ns dynamics in explicit water.

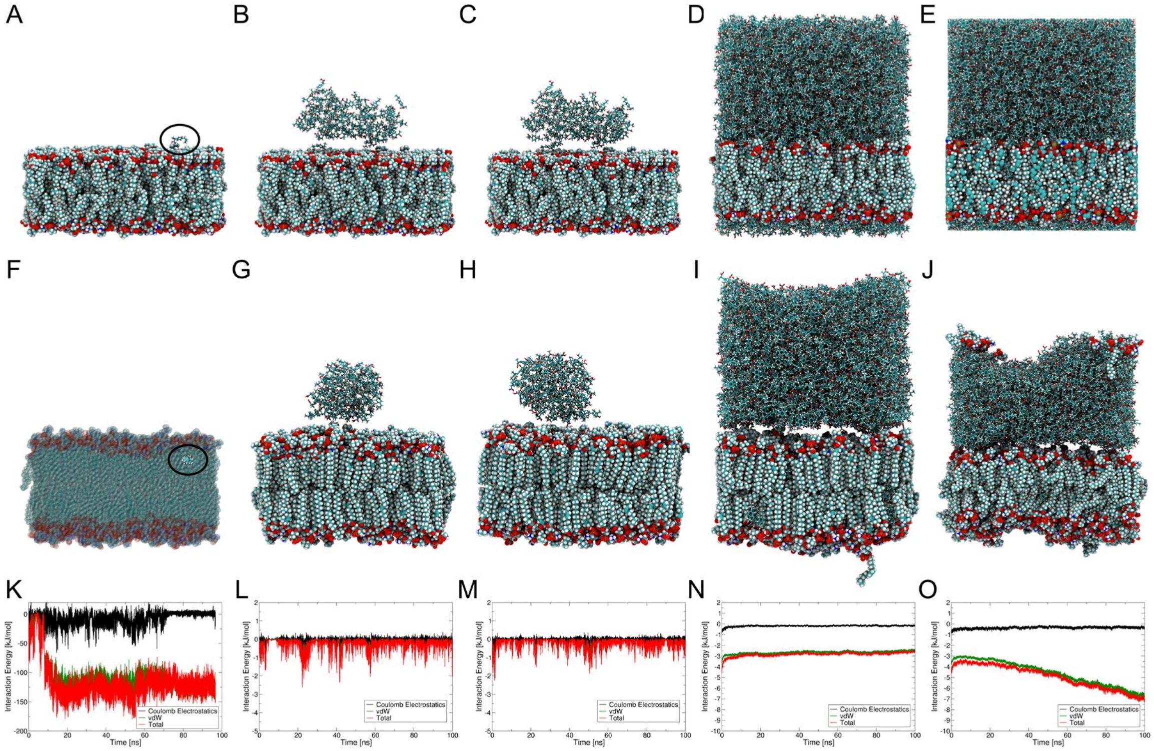

Fig. S13. *(A-E)* Initial and *(F-J)* final snapshots of *(A, F)* single ibuprofen molecule, aggregates of *(B, G)* 80 ibuprofen molecules, *(C, H)* 100 ibuprofen molecules, densely packed *(D, I)* 1903 ibuprofen molecules under NPT conditions, and *(E, J)* 1903 ibuprofen molecules under NVT conditions. *(K-O)* Ibuprofen–membrane interaction energies and their Coulombic electrostatic and van der Waals (vdW) components for all systems modelled in this study.

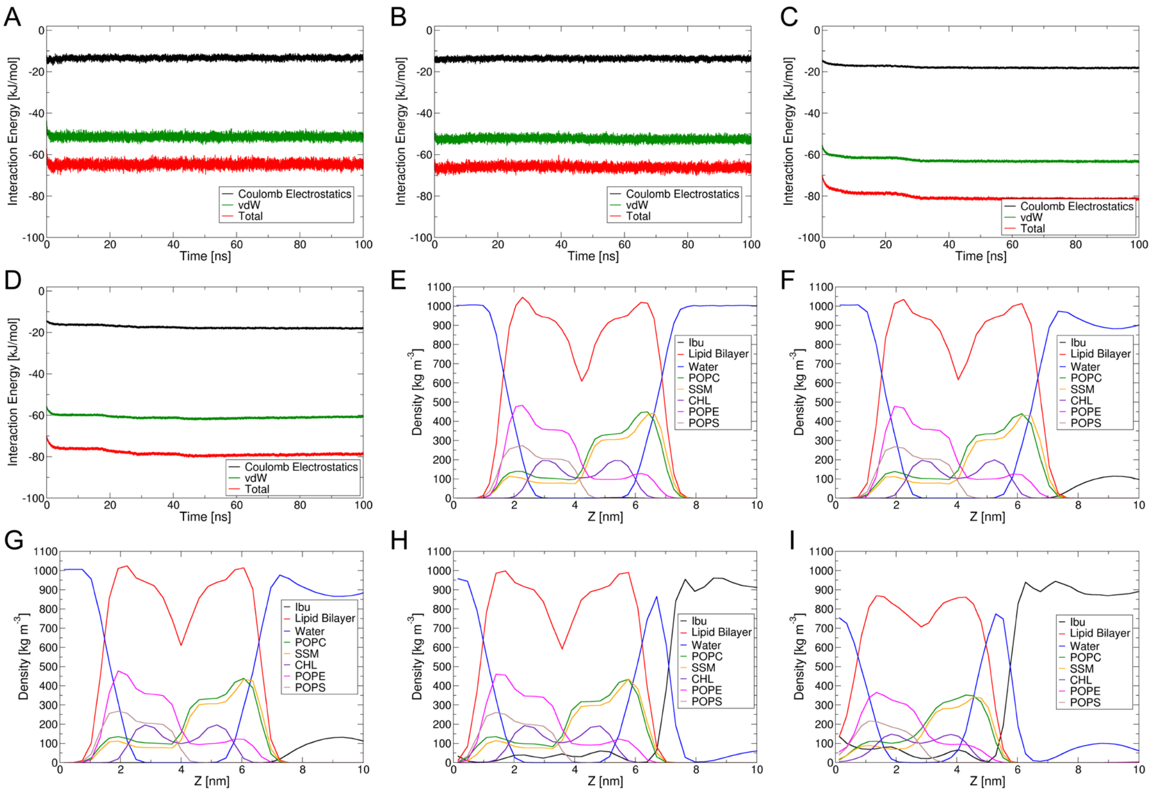

Fig. S14. Ibuprofen–ibuprofen interaction energies and their electrostatic and vdW components for *(A)* low conc. I, *(B)* low conc. II, *(C)* high conc. I, and *(D)* high conc. II (see Section S1.2 for these definitions). *(E-I)* Average density profiles of all species in the simulation box for all systems used in this study.

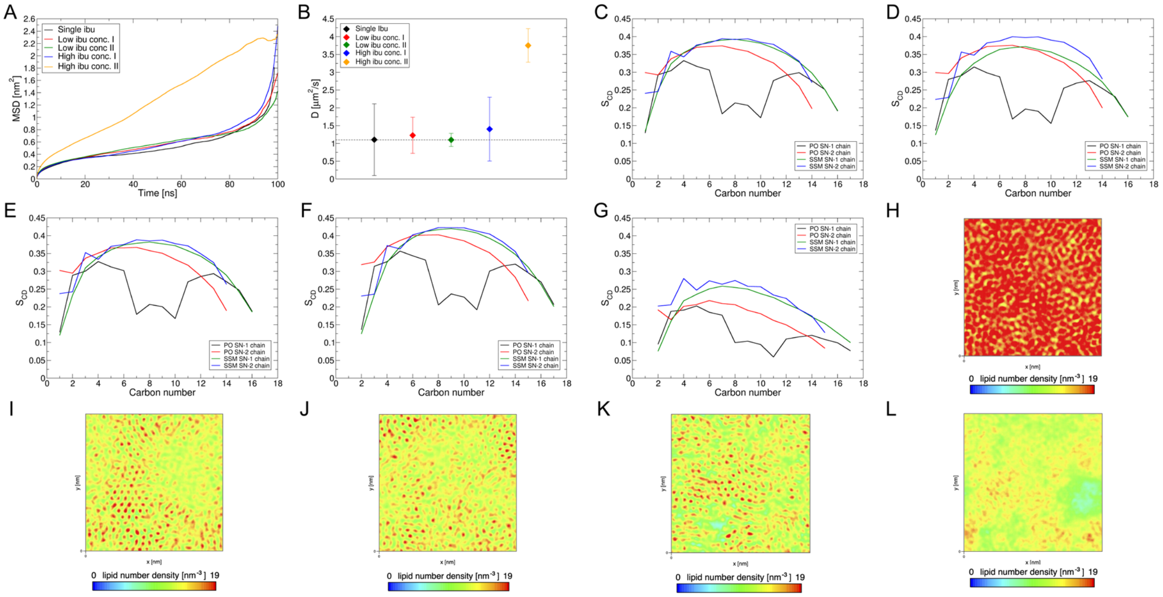

Fig. S15. Comparison of *(A)* Mean Square Displacement (MSD) and *(B)* diffusion coefficient, *D* for all systems modelled in this study. (C-G) Lipid hydrocarbon tail deuterium order parameter (S_{CD}) for RBC membrane lipids (Palmitoyl Oleoyl and Stearoyl) with two acyl chains, SN-1 and SN-2, and (H-L) lipid heavy atoms number densities in the *xy*-plane averaged over the *z*-axis to obtain a top view of lipid densities in the membrane for all systems.

Table S1. Summary of the demographics of the healthy blood and SCT and SCA RBC samples.

	**Gender**	**Age**	**Ethnicity**
**Healthy**	F	27	Caucasian
**SCT**	M	34	Caucasian
**SCA**	F	28	African American

Table S2. Overview of the total number of analyzed RBCs for the healthy, SCT and SCA samples and the percentages of each RBC type that was present in each sample.

	Total number of cells	RBC type								
		Normocyte	Stomatocyte	Echinocyte	Helmet	Acanthocyte	Spherocyte	Sickle	Ovalocyte	Teardrop
**Healthy**	351	272 (77.5%)	77 (21.9%)	1 (0.3%)	1 (0.3%)	0 (0%)	0 (0%)	0 (0%)	0 (0%)	0 (0%)
**SCT**	459	71 (15.5%)	0 (0%)	306 (66.7%)	1 (0.2%)	55 (12%)	25 (5.4%)	1 (0.2%)	0 (0%)	0 (0%)
**SCA**	230	62 (27%)	2 (0.9%)	122 (53%)	0 (0%)	23 (10%)	1 (0.4%)	10 (4.3%)	9 (4%)	1 (0.4%)

Table S3. Description of the extracted morphological parameters from Imaris 9.7.

**Morphological Parameter**	**Description**
Diameter	The length of the longest principal axis inside the object (*BoundingBoxOO Length C*)
Surface Area	The sum of the triangle surfaces
Volume	Quantification of how much a surface object occupies
S/V Ratio	Surface area divided by the volume
Thickness	The length of the shortest principal axis (*BoundingBoxOO Length A*)
Sphericity	The ratio of the surface area of a sphere to the surface area of the particle
Mean RI	Mean intensity of voxels enclosed within the surface

Table S4. Composition of RBC membrane lipid bilayer molecular model. CHL = Cholesterol, POPC = Palmitoyl Oleoyl PhosphoCholine, POPE = Palmitoyl Oleoyl PhosphoEthanolamine, POPS = Palmitoyl Oleoyl PhosphoSerine, and SSM = Stearoyl SphingoMyelin.

	CHL	POPC	POPE	POPS	SSM
**Inner leaflet**	40 (20%)	22 (11%)	76 (38%)	44 (22%)	18 (9%)
**Outer leaflet**	40 (20%)	70 (35%)	20 (10%)	0 (0%)	70 (35%)

Table S5. Overview of the ibuprofen concentrations and the corresponding ibuprofen dosage and ibuprofen plasma concentration.

	**Ibuprofen concentration**				
	0.25 mM	0.5 mM	1 mM	1.5 mM	3 mM
**Ibuprofen dosage**	200 mg	400 mg	800 mg	1200 mg	2400 mg
**Ibuprofen plasma concentration**	51 µg/mL	103 µg/mL	205 µg/mL	308 µg/mL	615 µg/mL

Table S6. Overview of the total number of analyzed RBCs for each ibuprofen concentration (0.25 mM, 0.5 mM, 1 mM, 1.5 mM and 3 mM), during a 20-minute time-lapse.

		Field of view 1	Field of view 2	Total
**Ibuprofen concentration**	**0.25 mM**	10	16	26
	**0.5 mM**	10	13	23
	**1 mM**	17	14	31
	**1.5 mM**	12	14	26
	**3 mM**	11	18	29

Movie S1. Effect of 0.25 mM ibuprofen on RBCs. RI tomograms were acquired at 2 sec intervals over a period of 20 min. 3D renderings of extracted frames from the video are provided in Fig. 4. Timecode is min:sec.

Movie S2. Effect of 0.5 mM ibuprofen on RBCs. RI tomograms were acquired at 2 sec intervals over a period of 20 min. 3D renderings of extracted frames from the video are provided in Fig. 4. Timecode is min:sec.

Movie S3. Effect of 1 mM ibuprofen on RBCs. RI tomograms were acquired at 2 sec intervals over a period of 20 min. 3D renderings of extracted frames from the video are provided in Fig. 4. Timecode is min:sec.

Movie S4. Effect of 1.5 mM ibuprofen on RBCs. RI tomograms were acquired at 2 sec intervals over a period of 20 min. 3D renderings of extracted frames from the video are provided in Fig. 4. Timecode is min:sec.

Movie S5. Effect of 3 mM ibuprofen on RBCs. RI tomograms were acquired at 2 sec intervals over a period of 20 min. 3D renderings of extracted frames from the video are provided in Fig. 4. Timecode is min:sec.

Movie S6. 3D segmented rendering of a single RBC exposed to low (0.25 mM) ibuprofen concentration and measured with DHTM, showing transient spicule formation, movement and dissolution across the RBC membrane. Timecode is min:sec.

Movie S7. 3D segmented rendering of a single RBC exposed to high (1.5 mM) ibuprofen concentration and measured with DHTM, showing irreversible spicule formation and movement across the RBC membrane. Timecode is min:sec.

SI References

1. R. Chan, M. Falato, H. Liang, L. Y. Chen, In silico simulations of erythrocyte aquaporins with quantitative in vitro validation. *RSC Adv* **10**, 21283-21291 (2020).
2. J. Lee *et al.*, CHARMM-GUI Membrane Builder for Complex Biological Membrane Simulations with Glycolipids and Lipoglycans. *J Chem Theory Comput* **15**, 775-786 (2019).
3. E. L. Wu *et al.*, CHARMM-GUI Membrane Builder toward realistic biological membrane simulations. *J Comput Chem* **35**, 1997-2004 (2014).
4. D. Van Der Spoel *et al.*, GROMACS: fast, flexible, and free. *J Comput Chem* **26**, 1701-1718 (2005).
5. K. Vanommeslaeghe, A. D. MacKerell, Jr., Automation of the CHARMM General Force Field (CGenFF) I: bond perception and atom typing. *J Chem Inf Model* **52**, 3144-3154 (2012).
6. K. Vanommeslaeghe, E. P. Raman, A. D. MacKerell, Jr., Automation of the CHARMM General Force Field (CGenFF) II: assignment of bonded parameters and partial atomic charges. *J Chem Inf Model* **52**, 3155-3168 (2012).
7. J. Huang *et al.*, CHARMM36m: an improved force field for folded and intrinsically disordered proteins. *Nat Methods* **14**, 71-73 (2017).
8. S. Boonstra, P. R. Onck, E. Giessen, CHARMM TIP3P Water Model Suppresses Peptide Folding by Solvating the Unfolded State. *J Phys Chem B* **120**, 3692-3698 (2016).
